# Direct Restorative Closure of Maxillary Midline Diastema Using a 3D‐Printed Matrix: A Case Report

**DOI:** 10.1155/crid/6396137

**Published:** 2026-05-03

**Authors:** Yu Toida, Shuhei Hoshika, Kenta Tsuchiya, Midori Kawamura, Chiharu Kawamoto, Atsushi Tomokiyo

**Affiliations:** ^1^ Department of Restorative Dentistry, Division of Oral Health Science, Graduate School of Dental Medicine, Hokkaido University, Sapporo, Japan, hokudai.ac.jp; ^2^ Department of Dental Medical Laboratory, Hokkaido University Hospital, Sapporo, Japan, hokudai.ac.jp

**Keywords:** 3D printing, anterior midline diastema, case report, digital dentistry, direct restoration

## Abstract

Anterior midline diastema is a common esthetic concern that can adversely affect smile appearance. Direct composite resin restoration has emerged as a conservative treatment option for diastemas associated with tooth size discrepancies, as it allows intraoperative adjustment of tooth morphology and shade. Digital dentistry has improved clinical outcomes by increasing workflow efficiency, enabling integration with other healthcare technologies, and supporting more accurate and predictable treatments. The objective of this case report was to describe the management of anterior midline diastema and spacing in the maxillary anterior region using a 3D‐printed matrix to achieve the intended esthetic outcome. An optical impression was acquired using an intraoral scanner. Because the occlusal relationship of the remaining dentition was stable, the maxillomandibular relationship was recorded using the same device. A maxillary incisal matrix was digitally designed using dental CAD software and fabricated with a 3D printer in a transparent photopolymerizable resin. After the matrix was positioned on the maxillary arch, a flowable composite resin was injected into the mold and subsequently light‐cured. Occlusal interferences were eliminated, and all restorations were finished and polished using polishing strips and a silicone point. Postoperative instructions were provided. The teeth were successfully reshaped in accordance with the digital design, achieving the planned esthetic and functional improvements. Direct composite restoration using a 3D‐printed matrix provides a rapid, convenient, and predictable approach for managing anterior midline diastema. This technique enables precise transfer of the digital design to the clinical setting, allowing improved control of morphology and symmetry. A 3D‐printed matrix can therefore be considered a reliable alternative to conventional freehand composite restoration for maxillary anterior esthetic rehabilitation, particularly in cases involving midline diastema and spacing.

## 1. Introduction

Anterior midline diastema represents a common esthetic concern and a form of malocclusion that can compromise smile attractiveness [1]. Evidence indicates that such spacing adversely affects patients’ esthetic self‐perception and perceived social acceptance [2]. Available management strategies include orthodontic, prosthetic, and restorative approaches [3, 4]. Restorative approaches achieve space closure by increasing tooth dimensions while maintaining the existing root position and can be carried out using composite resin or interproximal porcelain restorations [5, 6]. Indirect options, particularly ceramic veneers, are widely used for anterior midline diastema closure due to their durability and favorable long‐term clinical performance. However, these approaches often require additional tooth preparation. By contrast, direct techniques—mainly using composite resin—are less invasive and more readily repaired [7]. Contemporary composite resins can be shaped intraoperatively to achieve the desired morphology and shade, making direct composite restoration a suitable option for diastemas associated with tooth‐size discrepancies. Evidence indicates that closure of anterior midline diastemas using the injection technique with flowable composite resin yields stable and predictable outcomes [8]. Nevertheless, direct composite resin restorations are time‐consuming and highly technique‐sensitive, relying heavily on operator skill [9]. Even for experienced clinicians, achieving satisfactory tooth contours with a freehand approach remains challenging [10].

To overcome these limitations, digital tools and techniques have been introduced into restorative dentistry [11]. These innovations have improved accuracy and precision, enhanced the patient experience, increased efficiency and productivity, and facilitated more effective communication and collaboration among dental professionals [12, 13]. Technical developments have also improved clinical outcomes, streamlined laboratory workflows, enabled integration with other healthcare technologies, and supported more accurate diagnosis and treatment planning across dental specialties [14, 15]. These technologies extend beyond restorative dentistry to orthodontics, implant dentistry, and esthetic dentistry, enabling highly accurate and individualized treatment planning for patients [14, 16–19]. In restorative practice, direct composite resin restorations guided by 3D‐printed matrices have emerged as an efficient treatment option [10]. Simplified matrix‐based approaches have been proposed to reduce technique sensitivity, as the use of a matrix during composite resin placement streamlines the chairside workflow [6].

This study investigated a simplified direct composite resin restoration technique employing a modified injection matrix for anterior midline diastema closure and esthetic enhancement, integrating an intraoral scanner, CAD software, and 3D printing. Based on these considerations, we developed a novel composite injection matrix incorporating an interproximal isolation design to facilitate diastema closure using a 3D‐printed matrix. This article presents a case of anterior midline diastema and spacing in the maxillary anterior region managed using a 3D‐printed matrix to achieve the intended esthetic outcomes.

## 2. Case Report

### 2.1. Diagnostic Assessment

A 34‐year‐old female patient presented to the Department of Restorative Dentistry.

**Figure 1:**
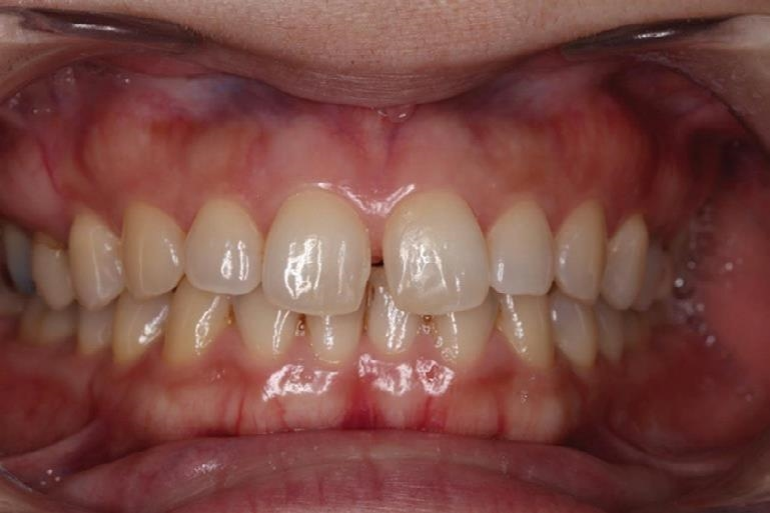
Initial frontal photograph before the restoration.

Her medical and dental history was unremarkable. In addition, no notable oral health issues were reported in her family. Clinical examination revealed healthy gingiva, a Class I molar interocclusal relationship, a deep overbite, and a maxillary midline diastema involving the anterior occlusion (Figure 1). After discussing three treatment options—direct composite resin restoration, indirect restoration, and orthodontic treatment—the patient elected to undergo direct composite resin restoration using a 3D‐printed matrix. Intraoral photographs were obtained for documentation and communication, and an optical impression was captured using an intraoral scanner (Medit i700, Medit Corp., Seoul, South Korea).

### 2.2. CAD Design Protocol

**Figure 2:**
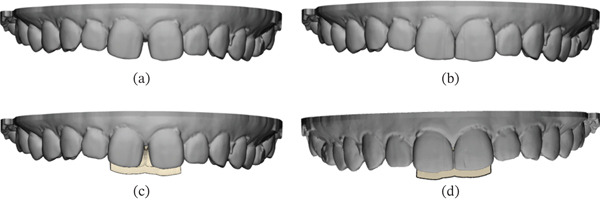
Buccal view of the digital intraoral scan showing the patient’s dental arch and matrix. (a) Buccal view of the dental arch prior to CAD design. (b) Buccal view of the digital wax‐up designed in CAD software. (c) Buccal view of the matrix try‐in without digital wax‐up. (d) Buccal view of the matrix try‐in, including the digital wax‐up.

**Figure 3:**
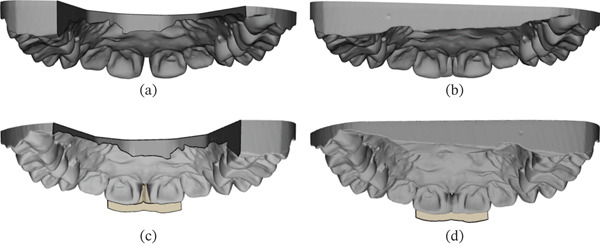
Palatal view of the digital intraoral scan showing the patient’s dental arch and matrix. (a) Palatal view of the dental arch prior to CAD design. (b) Palatal view of the digital wax‐up designed in CAD software. (c) Palatal view of the matrix try‐in without digital wax‐up. (d) Palatal view of the matrix try‐in, including digital wax‐up.

**Figure 4:**
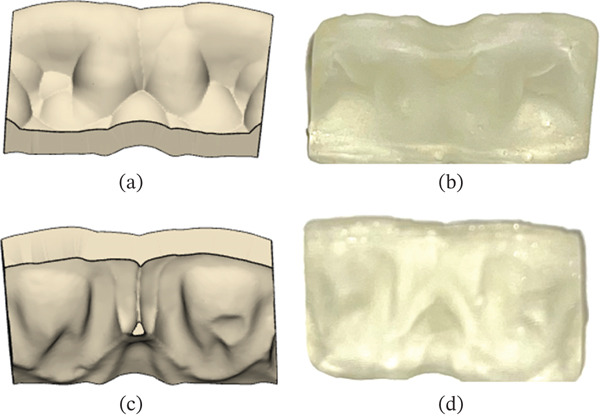
Design of the matrix. The matrix was designed using dental CAD software. (a) Buccal view of the CAD‐designed matrix. (b) Buccal view of the 3D‐printed matrix (c). Palatal view of the CAD‐designed matrix. (d) Palatal view of the 3D‐printed matrix.

The optical impression data was exported as an STL file and imported into CAD software (Dental System, 3Shape, Copenhagen, Denmark) (Figures 2 and 3). Margins were delineated on the digital model. A standardized digital wax‐up from the software library was selected and subsequently adjusted manually to achieve the desired morphology (Figures 2 and 3). The waxed‐up dental arch data were imported into CAD software (3‐matic Medical, Materialise N.V., Belgium). A matrix with a uniform thickness of 1.5 mm was generated from the waxed‐up dentition, trimmed at the distal aspects of both maxillary central incisors, and divided into buccal and lingual components. The final design was transferred to a 3D printer (Sonic Mighty 4K, Phrozen, Xiangshu, Taiwan) and fabricated using a transparent photopolymerizable resin (DH Print Splint & Guide, Denken‐Highdental, Kyoto, Japan) (Figure 4).

### 2.3. Direct Restorative Composite Application Protocol

Shade selection was performed under natural daylight with hydrated teeth using a VITA Classical Shade Guide (VITA Zahnfabrik, Bad Säckingen, Germany). A supra‐nano spherical filler–based light‐cured composite resin (shade A3, Estelite Flow Quick, Tokuyama Dental, Tokyo, Japan) was selected. A flowable composite was applied as a thin adaptation layer within the matrix, followed by a sculptable composite to achieve contour control.

The 3D‐printed matrix was tried in and evaluated for complete seating, stability, and accurate adaptation to the palatal surfaces. The planned incisal edge position and palatal contour were confirmed. A thin layer of petroleum jelly was applied to the internal surface of the 3D‐printed matrix. The maxillary left central incisor was isolated with polytetrafluoroethylene (PTEE) tape (TDV Isotape, Morimura, Tokyo, Japan).

The maxillary right lateral incisor was etched with 35% phosphoric acid gel (K‐Etchant Gel, Kuraray Noritake Dental, Tokyo, Japan) for 10 s and then rinsed with water. A primer and bonding agent (Clearfil SE Bond 2, Kuraray Noritake Dental) were applied to the enamel and light‐cured for 10 s using a wireless curing unit (Pencure 2000, Morita, Osaka, Japan) with a wavelength range of 360–540 nm and an intensity of 2000 mW/cm^2^. With the matrix sealed on the maxillary arch, flowable composite resin was injected and light‐cured for 10 s per increment. Each increment was carefully adapted to prevent voids and overhangs. Symmetry and midline alignment were assessed repeatedly during buildup.

**Figure 5:**
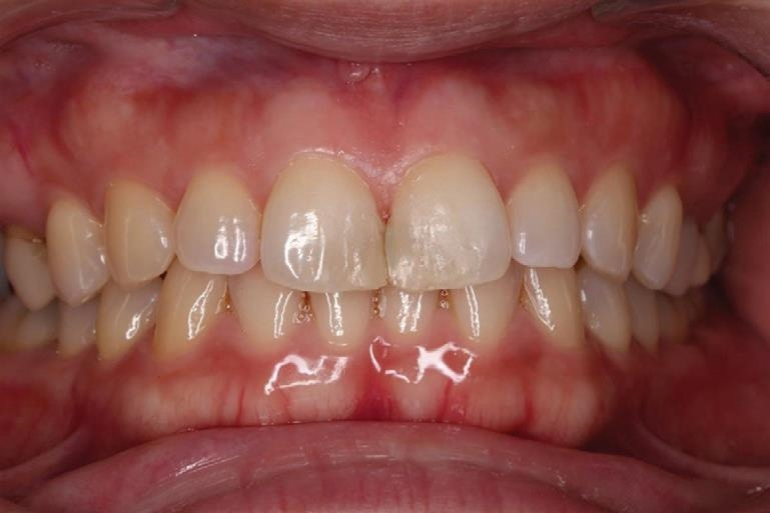
Final frontal photograph after restoration.

Occlusion was evaluated using articulating paper (Precut Articulating Paper, Morita, Osaka, Japan) in maximum intercuspation (static occlusion) and during guided mandibular lateral excursions (dynamic occlusion). After adequate polymerization, the matrix was removed. Excess resin was trimmed using fine diamond burs (Smooth Cut #B18ff, GC, Tokyo, Japan). The emergence profile and cervical adaptation were clinically verified. Final finishing and polishing were performed using polishing strips (New Metal Strips, GC) and a silicone polishing point (Compo Master, Shofu, Kyoto, Japan) to achieve smooth surfaces and natural luster. The teeth were reshaped according to the planned design, achieving the intended esthetic and functional outcomes (Figure 5). Postoperative instructions were provided.

### 2.4. Follow‐Up Assessment

At the 3‐month follow‐up, the restoration demonstrated excellent clinical outcomes. The patient maintained good oral hygiene and reported complete satisfaction with the outcome. Patient expectations and posttreatment satisfaction were recorded using a visual analogue scale; scores indicated full satisfaction both immediately after treatment and at the 3‐month follow‐up.

## 3. Discussion

Anterior midline diastemas in the anterior dentition can adversely affect smile esthetics and have psychological and social consequences [8]. These diastemas can be closed or reduced through orthodontic treatment, prosthetic approaches, restorative procedures, or combinations thereof [3, 4]. Direct composite resin restorations represent a conservative treatment option and are therefore preferred by many clinicians and patients [1]. Such restorations can be carried out freehand or with the aid of a polyvinyl siloxane matrix fabricated from a diagnostic wax‐up [20].

However, inherent challenges associated with direct techniques can compromise esthetics, marginal adaptation, and the quality of proximal contacts [21]. Proximal contours are commonly formed using a polyester strip and an interproximal wedge; however, this approach can hinder the establishment of a stable contact point and compromise marginal adaptation at the gingival margin of the proximal restoration [21]. These limitations not only reduce esthetic quality but may also increase the risk of periodontal complications, such as gingival inflammation due to bacterial biofilm accumulation or food impaction resulting from an inadequate contact point [22].

To address these issues, careful diagnosis and treatment planning are required, including identification of the etiologic factors underlying anterior midline diastemas. Recent advances in digital technologies—digital impressions, CAD, and 3D printing—have transformed these workflows and show considerable promise for improving direct restorative techniques [23]. The integration of digital tools improves the accuracy and predictability of direct composite resin restorations, enabling more precise and reproducible restoration morphology with improved safety [24]. Digital workflows also facilitate patient education and engagement, which is important given the subjective nature of esthetic outcomes and the need to align patient expectations with achievable results [25]. Preoperative visualization and discussion support informed treatment decisions and help establish realistic expectations, thereby reducing the risk of subsequent disputes.

A palatal silicone matrix derived from a preoperative mock‐up can reliably reproduce the anatomical form. In the present study, esthetic closure of an anterior midline diastema was achieved using a 3D‐printed matrix generated from an intraoral scan and CAD design. Interproximal walls were reconstructed using the 3D‐printed matrix rather than conventional mylar strips [26]. Currently, direct reconstructions using silicone or 3D‐printed guides represent viable options for closing anterior midline diastema [27]. A silicone build‐up guide is commonly used in the esthetic management of direct composite resin restorations, and a 3D‐printed matrix can serve the same function [27].

Digital workflows reduce laboratory time by eliminating conventional tray preparation, disinfection, the shipping of impressions, and the subsequent fabrication of gypsum casts [28]. CAD design eliminates the need for a separate mock‐up while improving accuracy and precision [28]. Once the design is finalized, the pattern can be 3D printed for both patient communication and fabrication purposes, such as illustrating the expected treatment outcome or preparing a tray on a 3D‐printed cast [10]. The CAD‐designed and 3D‐printed template restoration serves as a guide for the final restoration, and the precision of the mold is critical for achieving an accurate fit and a natural appearance [29]. To reduce costs, a hand‐splint tray was used instead of a clear silicone matrix; this approach was able to reproduce fine details, including contours and curvatures, at minimal expense. Due to the compact matrix design, closure of the maxillary midline diastema was achieved using only a palatal template, thereby reducing material consumption and minimizing environmental impact. Furthermore, in settings where it is not possible for clinicians to access specialized equipment or possess advanced technical expertise, the widespread adoption of this technique in routine clinical practice may be limited. However, several limitations and potential risks should be considered. The accuracy of the matrix depends on the quality of intraoral scanning, CAD design, and printing resolution; errors at any stage may lead to marginal discrepancies or inadequate contour reproduction [30]. It may also be limited in cases with severe malocclusion, large diastemas requiring orthodontic correction, or inadequate enamel for bonding.

Further research, particularly a well‐designed RCT with long‐term follow‐up, is needed to establish the reliability of the proposed technique.

## 4. Conclusions

Direct composite resin restoration of the maxillary central incisors using a 3D‐printed matrix represents a rapid, convenient, and predictable approach that achieves both esthetic and functional outcomes in the management of anterior midline diastema. This case demonstrates its feasibility and integration within a digital workflow.

## Author Contributions

Y.T.: conceptualization, methodology, investigation, data curation, formal analysis, visualization, resources, writing (original draft), and project administration. S.H.: conceptualization, methodology, validation, resources, and writing—review and editing. K.T.: validation and writing—review and editing. M.K.: software, validation, visualization, and writing—review and editing. C.K.: validation and writing—review and editing. A.T.: conceptualization, investigation, project administration, supervision, and writing—review and editing.

## Funding

No funding was received for this manuscript.

## Disclosure

All authors have read and approved the final version of the manuscript. Yu Toida had full access to all of the data in this study and takes complete responsibility for the integrity of the data and the accuracy of the data analysis.

## Ethics Statement

This report was approved by the Ethical Review Board for Life Science and Medical Research, Hokkaido University Hospital (No. 022–0176).

## Consent

Informed consent was obtained from the patient for the use of their medical data, images, and case details in this report. The patient agreed to have their anonymized information included in this case report.

## Conflicts of Interest

The authors declare no conflicts of interest.

## Data Availability

The data that support the findings of this report are available from the corresponding author upon reasonable request.
